# Evaluation Design for Community-Based Physical Activity Programs for Socially Disadvantaged Groups: Communities on the Move

**DOI:** 10.2196/resprot.2327

**Published:** 2013-06-26

**Authors:** Marion Herens, Annemarie Wagemakers, Lenneke Vaandrager, Johan Van Ophem, Maria Koelen

**Affiliations:** ^1^Chairgroup Health and SocietyDepartment of Social SciencesWageningen University and Research CentreWageningenNetherlands; ^2^Chairgroup Economics of Consumers and HouseholdsDepartment of Social SciencesWageningen University and Research CentreWageningenNetherlands

**Keywords:** community-based physical activity program, cost-effectiveness, low socioeconomic status groups, health promotion, evaluation design

## Abstract

**Background:**

As interventions are not yet successful in substantially improving physical activity levels of low socioeconomic status groups in the Netherlands, it is a challenge to undertake more effective interventions. Participatory community-based physical activity interventions such as Communities on the Move (CoM) seem promising. Evaluating their effectiveness, however, calls for appropriate evaluation approaches.

**Objective:**

This paper provides the conceptual model for the development of a context-sensitive monitoring and evaluation approach in order to (1) measure the effectiveness and cost-effectiveness of CoM, and (2) develop an evaluation design enabling the identification of underlying mechanisms which explain what works and why in community-based physical activity programs.

**Methods:**

A cohort design is proposed, based on multiple cases, measuring impact, processes, and changes at each of the distinguished levels. The methods described in this paper will evaluate both short- and long-term effects, costs, and benefits of CoM.

**Results:**

Testing of the proposed model began in October 2012 and is on-going.

**Conclusions:**

The design offers a valid research strategy for evaluating the effectiveness of community-based physical activity programs. Internal validity is guaranteed by the use of several verification techniques such as triangulation. The multiple case studies at the program and community levels enhance external validity.

## Introduction

### Background

Physical inactivity is one of the four core risk factors for non-communicable diseases such as diabetes type 2 and cardiovascular diseases. It has been identified by the World Health Organization (WHO) as the fourth leading risk factor for global mortality in 2012, causing an estimated 3.2 million deaths globally [1].

In the Netherlands, the Dutch Healthy Physical Activity Guidelines (NNGB) have been in use as a standard for monitoring physical activity behavior at population level since 1998. These guidelines set the norm for healthy daily physical activity for adults at a minimum of 30 minutes of moderate activity at least 5 days a week [2]. Research shows that physical activity levels of the Dutch adult population are rising, from 44% in 2000 to 62% in 2009 meeting the guidelines for healthy physical activity [3]. Adults spend on average 178 minutes per day in physical activity. Work, school, and domestic activities are the most important sources of physical activity.

Not all population strata, however, show this upward trend. The engagement of low socioeconomic status (SES) groups in sports and physical activity in the Netherlands remains lower than in high SES groups [4], despite various policies promoting community-based health and physical activity programs at the national, regional, and local level [5]. The neighbourhood is recognized as a setting in which to promote health and physical activity and to strengthen people’s responsibility for their own health and social participation [5-7].

As interventions have not yet been successful in substantially improving physical activity levels of low SES groups, it is a challenge to undertake more effective interventions [8]. In line with national policy objectives, the Netherlands Institute for Sports and Physical Activity (NISB) developed and disseminated a community-based program enhancing physical activity in inactive low SES target groups: the Communities on the Move (CoM) approach. The aim of CoM is to enhance physical activity levels of low SES groups, in order to contribute to social participation, quality of life, and life satisfaction of individual participants. Since 2003, CoM has been carried out by a variety of user organizations in 37 municipalities, reaching over 100 groups. Preliminary results of the program are promising. An expert panel of the Dutch Centre of Healthy Living has approved CoM as theoretically underpinned [9], but its effectiveness and cost-effectiveness have not yet been researched comprehensively.

Community-based interventions like CoM are grounded in both individual and community level theories [9,10], calling for appropriate designs to evaluate them at different impact levels [11]. To our knowledge, community-based physical activity programs have not yet been assessed comprehensively on both process and indicators for effectiveness at multiple levels. The aim of this paper is to provide the conceptual model for the development of a context-sensitive monitoring and evaluation approach in order to (1) measure the effectiveness, including the cost-effectiveness, of CoM, and (2) develop an evaluation design enabling the identification of underlying mechanisms that explain what works and why in community-based physical activity programs. The proposed research design is based on insights derived from the authors’ experiences in community-based health promotion programs [12-14].

### The Communities on the Move Approach

CoM targets inactive, low SES groups. CoM is a principle-based approach, enabling community-based physical activity interventions to be tailored to the needs and demands of target groups within specific local contexts. The objective is to identify, assess, and mobilize available resources for physical activity within the target group and their community. This requires a participatory approach in program development and implementation, involving different stakeholders including the target population in all stages of program planning, implementation, and evaluation [15,16]. CoM is linked to the *assets for health* concept [17]—a health asset being any factor that enhances the ability of individuals, communities, populations, and/or social systems to improve or maintain health and well-being. The concept includes a salutogenic perspective on health, focusing on positive health outcomes [18,19].

The key principles of CoM, identified and used in a 4-year pilot phase (2003–2007), at the program and community levels are: intersectoral collaboration, coordinated action for sustainability, and active participation of local stakeholders (organizations and community representatives). The key principles at the group and individual levels are: a social network approach, active participation of participants in program development, enjoyment, group bonding, and creating supportive environments. Phase 1 of a CoM program starts with *problem definition*, based on community assessments identifying stakeholders, physical activity needs, and assets. Phase 2 is *planning and development* of program activities with local stakeholders, setting goals, and defining actions within contextual boundaries. Phase 3, the actual *implementation* phase, is a stepwise approach, starting with activities for *recruitment*. First, the participants are recruited by accessing community groups and mobilizing their social networks, where a community group may be women visiting a mosque, for instance. The second step is defining and implementing the *action* program using group members’ input to tailor physical activities to their needs. The third step is consolidation. Group members practice what they have learned and actively involve their social and physical environments in order to sustain their behavior change. Phase 4 of CoM is program *evaluation* to document impact and lessons learned for further dissemination. Table 1 is a schematic representation of a local CoM program.

**Figure 1:**
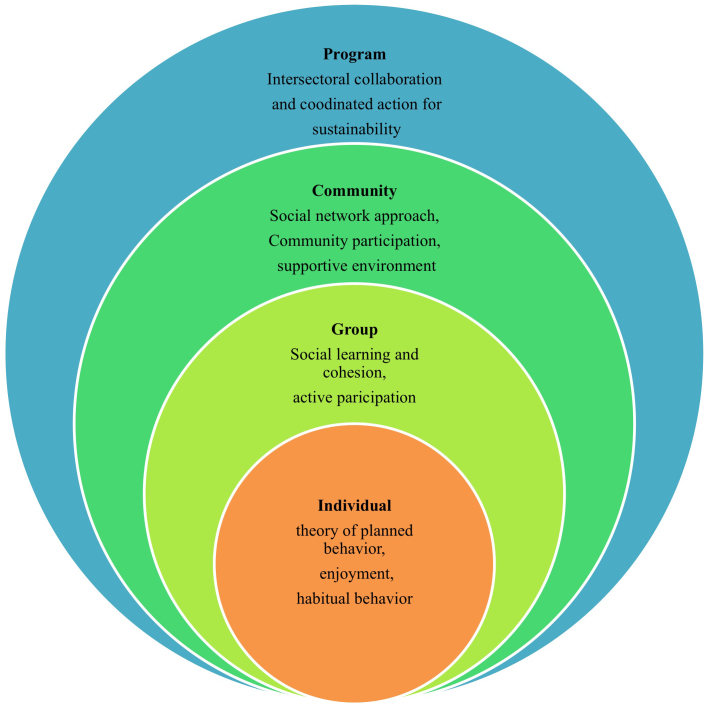
Theoretical underpinning of CoM.

Theories to develop and implement CoM use an ecological perspective on human health. The ecological perspective emphasizes the interaction between factors within and across the different levels [20]. To address the reciprocity of human interactions with their social and physical environment, CoM advocates actions at multiple levels, whereas each level builds on different theoretical frameworks (Figure 1). At the *individual* level, CoM aims to initiate and sustain change in physical activity behavior, building on the concepts of the theory of planned behavior (TPB). These concepts include behavioral intention, attitude, subjective norms and social influence, and self-efficacy [21]. CoM stimulates adherence to physical exercise and the development of habitual behavior through enjoyment [22-24]. At the *group* level, social learning processes and active participation, based on concepts of social cognitive theory (SCT), are used to support sustained behavioral change [20,25]. At the *community* level, CoM is based on the social network approach, community participation, and the notion of supportive environments. Social networks contribute to health [26] and effectively support physical activity behavior [27]. Community participation fosters higher levels of motivation and determines effectiveness [12]. At the *program* level, CoM is underpinned by theories on intersectoral collaboration and coordinated action [13], addressing stakeholder involvement and community ownership. Intersectoral collaboration strengthens the development and contextualization of the intervention by assessing assets and resources of various stakeholders and translates them into customised program activities. Intersectoral collaboration also contributes to the sustainable implementation of CoM.

**Table 1:** Principle-based CoM approach in local practice.

		Phase 1 Problem identification	Phase 2 Program development		Phase 3 Program implementation			Phase 4 Evaluation
				Recruitment	Action	Consolidation		
**Program organization**								
	*Intersectoral* *collaboration*	assessment community needs and assets	setting goals	program coordination and monitoring	program coordination and monitoring	program coordination and monitoring		formation new groups
		stakeholder involvement	program development	communication	communication	communication		
	*Program* *sustainability*	assessment policy goals	organizing resources	introduction activity program	physical activities program	physical activities program		
			capacity building	demonstration lessons	theme sessions			
**Community**								
	*Active participation*	identification target groups	identification key persons					formation new groups
	*Social network*			mobilizing participants				
	*Create supportive* *environments*					involving social and physical environment		
**Group**								
	*Group bonding*			getting acquainted	social learning	group cohesion		sustained group activities
	*Active participation*			program attendance	program attendance	group initiatives		
	*Create supportive* *environments*					involving social and physical environment		
**Individual**								
	*Social network*			participants acquainted		involving social and physical environment	sustained physical activity behavior	
	*Active participation*			assessment physical activity needs and ambitions	competence development (attitude, knowledge, skills)	competence development		
	*Pleasure*			exploring which physical activity are liked	learning to enjoy physical activity	competence and confidence development		

### Evaluation Objectives

CoM’s evaluation approach aims to (1) assess the effectiveness of CoM at different impact levels (individual, group, program, and community), (2) identify underlying mechanisms to explain the context sensitivity of program development and implementation, and (3) assess the cost-effectiveness of CoM. This paper will address the following research questions:

1. Which effects can be documented with respect to physical activity behavior, health, quality of life, and life satisfaction?
2. Which mechanisms explain the successes and failures of CoM in low SES groups and how can these be addressed?
3. How can results be interpreted in terms of costs and benefits and what combination of economic evaluation methods and tools is most appropriate to evaluate a community-based program on cost-effectiveness?

## Methods

### Study Design

**Figure 2:**
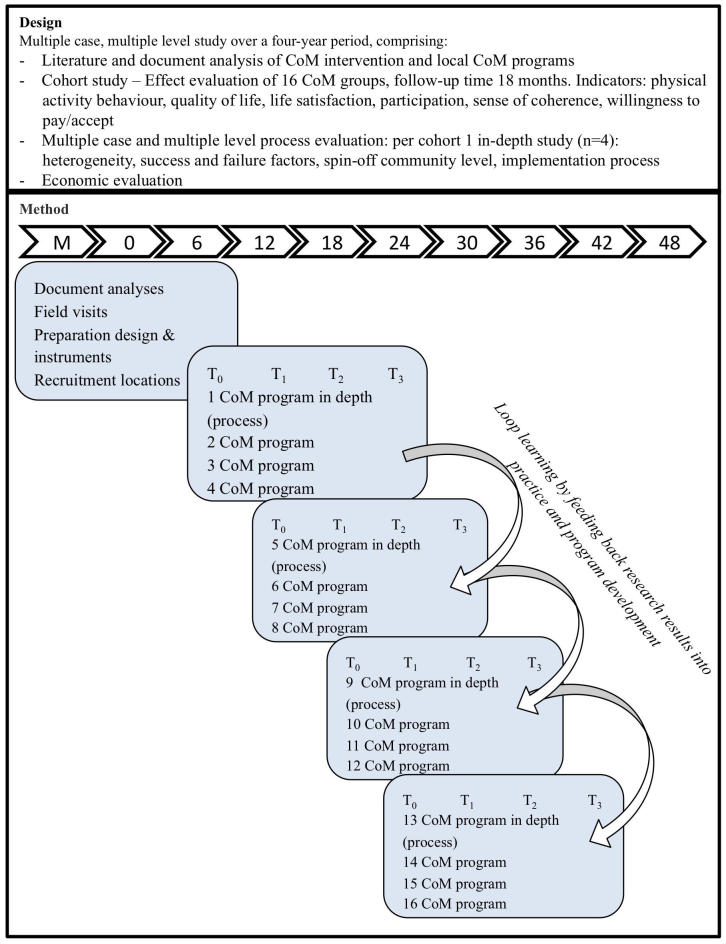
Evaluation design of CoM.

To measure the effectiveness and cost-effectiveness of CoM, our study combines a cohort analysis based on multiple cases, and a process evaluation and action research, measuring processes and changes at each of the 4 defined impact levels at multiple points in time (Figure 2). The study includes 16 groups of CoM programs in different municipalities, in 4 cohorts of 4 groups. Data will be collected through standardized questionnaires, open interviews, document analysis, interactive procedures, and focus groups. Four CoM programs (one case from each cohort) will be studied in depth. The advantage of a cohort analysis with cohorts starting successively over a course of 2.5 years is that multiple intermediate outcomes can be studied simultaneously over a period of time. It allows control for possible history and maturity effects, and as such it offers a valid alternative for a randomized controlled trial (RCT) design. RCT designs are considered less appropriate to assess the cost and effectiveness of CoM at multiple levels and to identify underlying mechanisms explaining success and failures for the following reasons [14,28]:

1. RCT designs focus on behavior change at individual and community population level, not taking into consideration conditions for change related to social, cultural, and organizational factors [14,29].
2. Applying the RCT design is difficult because of the absence of appropriate ways to define control groups in real life settings. Community-based physical activity promotion settings are generally open to the public at large, and people living in the control areas have access to the activities as well, hence, participants cannot be assigned randomly. Initial physical activity motivations for members of the community may also be different, making randomization difficult [14,28].
3. There are limitations in the ability of RCT designs to grasp the importance of interactions between the individual and his or her social and physical environment [30,31].

A mixed method design is therefore required to gain insight into the effectiveness of CoM programs at all 4 defined impact levels and to understand the process, the interactions and the quality of interactions needed for success [14,30]. An action approach enables researchers and local CoM stakeholders, including CoM participants, to apply and benefit from loop learning [12,32]. Learning loops are applicable to the CoM programs and to the overall learning processes of CoM and this research project. For local CoM programs, single-loop learning results in an improved local program. Double-loop learning results in adaptation of the organization of the program. The learning outcomes in the first four CoM programs can be used in the next four CoM programs and so on. As a consequence, during the research, CoM quality will be improved.

### Study Population

To assess outcomes at the *individual* and *group* levels, inclusion criteria for the study participants in CoM programs are inactivity, adults not meeting the NNGB, and low socioeconomic status (income, education, and employment conditions). In each CoM program, one or more groups will be included in the study for convenience sampling. During the study, 16 groups will be studied, each group consisting on average of 15 participants. Consequently, a total of 240 participants will be included. Data will be collected at 4 time-points: at the start of a local program (T_0_), at 6 months (T_1_), at 12 months (T_2_), and at 18 months (T_3_).

At *program* and *community* levels, on-going CoM programs will be included, based on existing partnerships between NISB and implementing organizations (purposive sampling). The study population consists of local stakeholders such as user organizations and networks in place, the disseminating organization (NISB), and community representatives.

### Logic Model

**Figure 3:**
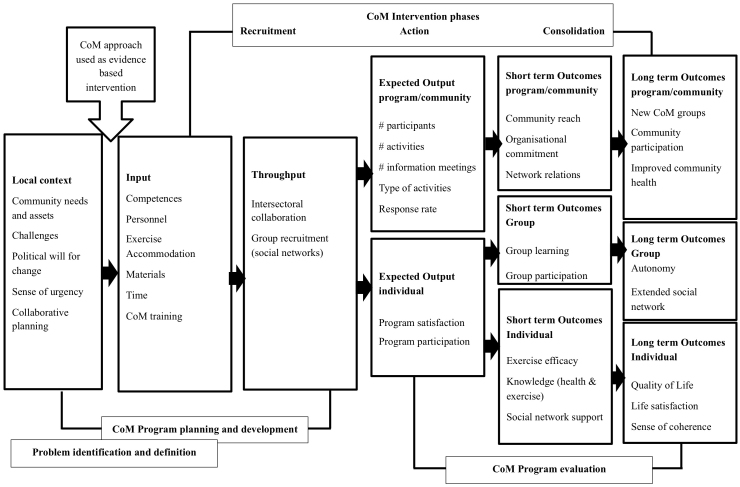
Logic model for evaluation effectiveness CoM.

Figure 3 illustrates the logic model for impact evaluation of CoM, based on the literature on community-based evaluation approaches [33] as well as dissemination studies of evidence-based interventions [34,35]. The hypothesis is that a community-based participatory approach to developing and implementing physical activity programs is effective in enhancing physical activity levels in low SES target groups and results in increases in quality of life, life satisfaction, and community participation.

The framework was developed based on the perspectives of the local program initiators and the community. Local program initiators seek the evidence base, developed in CoM and disseminated by NISB, whereas community-based approaches follow non-linear pathways of development and implementation [33]. This calls for process evaluation, addressing intersectoral collaboration, capacity building and network development, as well as identification of intermediate measures to be monitored at the different impact levels. *Short term output* is defined in terms of concrete activities, reach, and program satisfaction*. Short term outcome* indicators are defined in terms of measurable impact, such as increase in physical activity and knowledge, and the use of qualitative data (group learning) to understand outcomes. *Long term outcome* indicators are defined to measure broader outcomes and monitor local change.

### Impact Assessment

To assess effects with respect to physical activity behavior, quality of life, and life satisfaction at the *individual* level, a standardized questionnaire will be used to measure quantitative short- and long-term outcomes (Table 2). The questionnaire has been developed using concepts from the underlying theories of TPB, in addition to questions related to sports and physical activity behavior. Data on socioeconomic indicators will be collected (ie, age, income, education, employment, living conditions), in accordance with standardized questions in the Local and National Monitor Public Health in the Netherlands [36].

To measure physical activity, the validated Short QUestionnaire to ASses Health enhancing physical activity (SQUASH) will be used [37]. Correlations for reproducibility of the separate questions vary between 0.44 and 0.96. Spearman’s correlation coefficient between CSA readings and the total activity score was 0.45 (95% CI 0.17-0.66) [38]. The SQUASH questionnaire was used as it generates data that can be compared with national and regional data. The Dutch trend analyses for physical activity behavior over the past 2 decades were based on the SQUASH, offering a vast body of reference data for our study [3].

In this study we will explore the use of objective measures for physical activity, such as walking tests or accelerometers [39,40]. These objective measurements, however, generally require additional data such as generated by SQUASH, to be able to interpret outcomes on physical activity behaviors and the development of habitual physical activity behavior. Some challenges remain with the use of objective physical activity measures. First, validity and reliability can be questionable, for example, when these measures are used with user groups suffering from chronic diseases [41]. Second, organizational efforts and costs are practical issues related to implementation that must be considered [40].

To measure personal goals on health and physical activity behavior, a number of personal features will be documented (eg, demographics, BMI). To measure life satisfaction, Cantril’s Self-Anchoring Ladder for Life Satisfaction will be used [42]. To measure the ability to cope with stressors, the validated 13-item Sense of Coherence (SOC) questionnaire will be used [43]. Cronbach alpha values in 127 studies using SOC-13 ranged from 0.70 to 0.92 [44]. To measure enjoyment, the 9-item short version of Physical Activity Enjoyment Scale (PACES) will be used [45,46].

To assess mechanisms explaining successes and failures of CoM in low SES groups and how these can be addressed, data will be collected at the *group* and *program* level through interviews, focus groups, and document analysis (Table 2). A combination of action research and realism evaluation will be used. Action research is important because it has both an action function, which supports the progress of the intervention, and an evaluation function, which seeks to monitor and ascertain processes and outcomes of interventions [47]. Realism evaluation facilitates the study of the interactions between context and program mechanisms determining the outcomes [48]. To assess CoM’s context-based information, each of the CoM programs will include an interview with the program coordinator and the two focus groups—the local stakeholders and the CoM participants. To measure effectiveness at the *program* level, we will incorporate the factors for achieving and sustaining participation and collaboration [49], the coordinated action checklist [50], and Pretty’s participation ladder [25]. The RE-AIM dimensions (ie, reach, effectiveness-adoption, implementation, and maintenance) will serve as the framework to measure spin-offs and highlight areas that require special attention with respect to sustainability [51].

**Table 2:** Overview of variables and methods of data collection.

Level	Variables	Questionnaires				Document analysis	Interview	Focus group	Instruments
		T_0_	T_1_	T_2_	T_3_				
**Individual**									
	Age, gender, income, education, ethnic background	x							Questionnaire
	Quality of life	x	x	x	x				EQ-VAS
	Life satisfaction	x	x	x	x				Cantril’s ladder
	Physical activity and health behavior	x	x	x	x			x	Questionnaire
	BMI	x	x	x	x				Questionnaire
	Sense of Coherence	x			x				SOC-13 scale
	Enjoyment	x	x	x	x			x	PACES scale
	Willingness to pay	x	x	x					Questionnaire
	Personal goals	x	x	x	x			x	Questionnaire
**Group**									
	Social support	x	x		x			x	Questionnaire
	Participation							x	Timeline Pretty’s ladder
**Program**									
	Organization and collaboration					x	x	x	Coordinated action checklist
	Program participation	x	x				x	x	Pretty’s ladder
	Support and training					x	x	x	
	Competences						x	x	
	Diffusion		x			x	x	x	
	Cost per QALY	x	x	x					
	Cost-effectiveness		x			x			QALY
**Community**									
	Spin-off: new programs and community participation			x	x	x	x	x	RE-AIM framework

### Economic Evaluation of CoM

To assess how results can be interpreted in terms of costs and benefits and what combination of economic evaluation tools is most appropriate to evaluate a community-based program on cost effectiveness, results from the cohort analysis, process evaluation, and action research at all levels discerned will be used (Table 2). The study perspective in evaluating CoM’s cost-effectiveness will be the societal perspective. Data will be collected about health-related quality of life in relation to the physical activity program and its program costs over a time frame of 18 months. To measure health-related quality of life, the Dutch EuroQoL (quality of life) scale (EQ-5D-3L) and the EQ visual analogue scale will be used. The EuroQoL scale is standardized, measuring non-disease specific health-related quality of life, in use for economic evaluation [52,53].

The methods used will include traditional measures such as cost utility, cost-benefit analysis, Quality Adjusted Life Year (QALY, expressed in euros per quality-adjusted life-year) gained, and willingness to pay or accept. We will also use instruments that measure changes in life satisfaction and sense of coherence (SoC).

At the *individual* level, the most usual means to measure changes in welfare are compensation tests. Compensation tests, such as willingness to pay, are measured based on monetary value [45]. Willingness to pay questions (for sport and physical activity) will be asked at distinctive points in time during the CoM program. To measure health gain, the QALY will be calculated by multiplying the amount of time in a particular health state by the quality of life during that time, summing over all time periods, standardized to a year [54].

A cost-effectiveness analysis at the *program* level will be performed by computing cost per QALY gained. At program level, costs such as salaries, training costs, and materials are summed up, and benefits are measured through the computation of QALY gained at various time-points, as described above. The outcomes of these computations will be compared with other relevant interventions. In all methods applied, assumptions used in the economic calculations and evaluation will be made explicit.

### Analysis

#### Qualitative Analysis

Qualitative research data from interviews and focus group discussions will be audiotaped (with the interviewees’ permission), transcribed (intelligent verbatim style), and analyzed using Atlas.ti (version 7.0) to manage the data and guarantee transparency. Top-down as well as bottom-up coding will be used to provide for the analysis of differences in perspective of CoM participants, professionals, and scientists [55,56]. Case study data will be used to describe general mechanisms of failures and successes of the CoM program for various low SES groups.

#### Quantitative Analysis

Quantitative data will be analyzed with multivariate analysis techniques using the SPSS program. The quantitative variables at the individual level (Table 2) are to be tested for 4 independent variables (gender, age, ethnicity, and SES) using a multiple regression analysis with a significance level of .05 and a power of 0.80 for a medium effect size. This requires 84 participants for the study [57]. If there are several different groups (eg, ethnicity, SES) each with eight independent variables, 107 participants will be needed. Targeting 240 COM participants would satisfy these conditions.

#### Power Calculation

As the study design lacks control groups and consequently limits randomization, the assumption made in the power calculation is, that the CoM principles used are the same in each location. Effect sizes, therefore, can be calculated based on the overall population included in CoM programs.

The power calculation of the effectiveness of the CoM program is based on the variable *physical activity*, as the prime aim of the CoM program is to enhance physical activity in inactive, socially disadvantaged groups. Measures for change to be considered include: increase in the average number of minutes people are physically active, in the number of people meeting the Dutch Healthy Physical Activity Guidelines (NNGB), and in the number of people indicating that they are more physically active after participation in a CoM program.

Estimation of the effect size is based on an American systematic review study [27]. This review showed that the average time spent on physical activity increased by 35.4% (range 16.7-83.3%), based on 17 studies involving middle-aged adults. Dutch studies reviewing physical activity interventions gave no numerical information about effect sizes [58,59]. One intervention report showed an increase of 38% on average in the physical activity pattern. Based in these data, the estimated effect size for our study is set at an increase in physical activity of 35% in each group, roughly equivalent to 500 minutes a week.

A limitation of the proposed cohort design is the ability to correct for history or maturity effects, as the timeframe for data collection per cohort is restricted to 18 months with measurement intervals of only 6 months. To control for these effects, a comparison of cohorts will be conducted. Furthermore, comparisons will be made with existing population statistics for physical activity.

### Management and Governance

Research activities will be developed and implemented in close collaboration with NISB to stimulate active knowledge exchange and co-creation of new knowledge. In this way, so-called context-sensitive evidence will be generated, which by its nature is relevant for intended users [60].

For the research project, a steering group consisting of representatives from Wageningen University and NISB will meet regularly. In addition, advisors from national and international organizations (eg, the Dutch Centre of Healthy Living, other universities, and community programs) will be involved for specific purposes, such as to review the developed questionnaires, to critically assess results of the interviews and focus groups, and to comment on drafts of scientific articles.

### Intended Outputs

This study will result in recommendations for improving the health of low SES groups through physical activity. Further research results include:

1. An elaborated monitoring and evaluation design for participatory community health and physical activity promotion.
2. Assessment of CoM effectiveness and cost-effectiveness at the *individual*, *program*, and *community* levels.
3. The facilitation of wider implementation of CoM at both national and local level.

## Results

The study began in October 2012 with data collection at both the *individual (*T_0_) and *program* levels. Baseline data was collected for the first cohort. At the *program* level, documentation is collected and interviews are conducted with local stakeholders. The study is on-going and funded by ZonMw, the Netherlands Organization for Health Research and Development (project number: 50-51505-98-103).

## Discussion

### Need for an Alternative Evaluation Approach

The need to elaborate an alternative evaluation approach to study the effectiveness and cost-effectiveness of a community-based physical activity program such as the CoM is evident. New indicators, methods, and tools are required in a real-world setting, comprising multiple levels. The design described in this paper offers a valid research strategy for effectiveness, combining cohort analysis, process evaluation, and action research within multiple cases (parallel investigations in different settings), addressing the different impact levels in a comprehensive way.

Credibility or internal validity is guaranteed by the use of several verification techniques such as triangulation, stakeholder checking, external auditing, and peer review [31,61]. Triangulation of data obtained by questionnaires, interviews, and focus groups elucidates why effects on physical activity behavior, health related quality of life and life satisfaction have occurred.

The multiple cases carried out at the program and community level (4 in-depth cases) will enhance external validity. The findings of the study will be context specific and specific to different low SES groups, but will also reveal generic mechanisms of change.

### Value for Science, Practice, and Society

Conducting comparable studies in different situations will make it possible to draw conclusions about the quality of achievements and the processes and mechanisms in force in community-based projects, but also about the usefulness of (new) research techniques [31,47].

Practice will benefit from the research in various ways. Research activities will be part of the intervention, and stakeholders will participate in the development, implementation, and evaluation of research activities. Results will be fed back into the program immediately in order to undertake subsequent action. In addition, this research project will facilitate wider implementation of CoM.

Information on the effectiveness and cost-effectiveness of community health promotion is highly relevant for policy makers to decide on the implementation of community-based approaches. In view of the increasing number of programs expected as a result of Dutch health policies aiming at self-mobilization and organization in neighbourhoods, this study will address the need to contribute to insight into context-sensitive intervention development targeting low SES people who are physically inactive, and how to monitor and evaluate these in a comprehensive way.

## Abbreviations

BMI: body mass index
CoM: Communites on the Move approach
EQ-VAS: standard visual analogue scale for rating health-related quality of life
NISB: Netherlands Institute for Sports and Physical Activity
NNGB: Dutch Healthy Physical Activity Guidelines
PACES: Physical Activity Enjoyment Scale
RE-AIM: reach, effectiveness-adoption, implementation, maintenance
RCT: randomized controlled trial
SES: socioeconomic status
QALY: Quality Adjusted Life Year
SOC: sense of coherence
TPB: theory of planned behavior
SCT: social cognitive theory
SQUASH: Short QUestionnaire to ASses Health enhancing physical activity
QoL: quality of life
WHO: World Health Organization
ZonMw: Netherlands Organization for Health Research and Development
